# Activating KIR/HLA complexes in classic Kaposi's Sarcoma

**DOI:** 10.1186/1750-9378-7-9

**Published:** 2012-04-02

**Authors:** Franca R Guerini, Roberta Mancuso, Simone Agostini, Cristina Agliardi, Milena Zanzottera, Ambra Hernis, Athanasia Tourlaki, Maria G Calvo, Monica Bellinvia, Lucia Brambilla, Mario Clerici

**Affiliations:** 1 Don C. Gnocchi Foundation ONLUS, P. le Morandi 6, 20121 Milan, Italy; 2 Department of Biomedical Sciences and Technologies, University of Milan, Via F.lli Cervi 93; 20090 Segrate (Milano), Italy; 3 Dermatology Unit, IRCCS Ca' Granda Foundation - Ospedale Maggiore Policlinico, Milan, Italy

**Keywords:** Kaposi's Sarcoma, Human Herpesvirus 8, KSHV, NK cells, KIR, HLA

## Abstract

**Background:**

Classic Kaposi's Sarcoma (cKS) is a rare vascular tumor associated with Human Herpesvirus 8 (KSHV) infection, nevertheless not all KSHV-infected individuals have cKS.

**Objective:**

We investigated whether particular KIR/HLA receptor/ligand genotypes would be preferentially present in KSHV-infected and uninfected individuals who have or have not developed cKS.

**Methods:**

KIR/HLA genotypes were analyzed by molecular genotyping in 50 KSHV-infected individuals who did or did not have cKS and in 33 age-and sex-matched KSHV seronegative individuals.

**Results:**

There was no association of individual KIR, HLA or receptor ligand combinations with KSHV infection. However, activating KIR and KIR/HLA genotypes were significantly more frequent in cKS cases, specifically KIR3DS1, KIR2DS1, and KIR2DS1 with its HLA-C2 ligand.

**Conclusion:**

A nonspecific inflammatory response triggered by activation of NK cells upon KIR-HLA interaction could be associated with the pathogenesis of KS.

## Findings

Classic Kaposi's Sarcoma (cKS) is a rare vascular neoplasm of the skin related to Kaposi's Sarcoma-associated Herpes Virus (KSHV or Human Herpes Virus 8, HHV-8) infection. KSHV plays a prominent role in the progression of cKS from an angio-proliferative disorder to sarcoma, KSHV infection is nevertheless not sufficient to provoke such progression as not all KSHV-infected individuals develop cKS [1]. Natural killer (NK) cells are central components of the innate immune response against viral infections and tumour growth via direct and indirect mechanisms [2]. The modulation of NK activity is a complex and multi factorial phenomenon triggered by the binding of inhibitory or activating killer cell immunoglobulin-like receptors (KIR) to class I human leukocyte antigens (HLA) [3,4]. When HLA molecules bind activating KIR receptors, a potent inflammatory response finalized at NK cell-mediated destruction of target cells, including transformed tumor cells and virus-infected cells, is stimulated [5]. KIR and HLA loci are highly polymorphic and map in distinct human chromosomes (chromosomes 19 and 6, respectively); both KIR and the specific HLA ligands must be present in order to regulate NK cell activity, such that one without the other is functionally inert.

We verified whether particular KIR/HLA genotypes would be preferentially present in KSHV-infected and uninfected individuals who do or do not develop cKS.

We studied eighty-three Caucasian individuals born and living in Northern Italy; 50 of these individuals were KSHV-infected (KSHV^pos^). Histologically-confirmed cKS had been diagnosed in 32 patients (cKS^pos^)(23 male, 9 female, mean age: 71.1 ± 7.7 years); KSHV-infection without tumour was observed in 18 other patients. (KSHV^pos^/cKS^neg^)(8 male, 10 female, mean age: 83.4 ± 11.6 years). Thirty-three KSHV-uninfected individuals (KSHV^neg^)(24 male, 9 female, mean age: 70.0 ± 12.6 years) were also enrolled. Subjects did not suffer from any disease that could potentially bias the analysis outcome. KSHV-infected patients were enrolled, upon signing an informed consent approved by the Institutional Review Board, by the Dermatology Unit of the Ospedale Maggiore Policlinico, in Milan. Controls subjects were elderly healthy individuals seen at the Don Gnocchi Foundation in Milan.

We analyzed antibodies against latent and lytic KSHV antigens using an in-house immunofluorescence assay (IFA) based on the body-cavity B cell lymphoma (BCBL-1) cell line [6] as previously described [7]. Monoclonal antibodies to ORF73 (LNA-1), ORF 59, and ORF K8.1 lytic proteins (Advanced Biotechnologies Inc., Maryland, USA) were utilized as fluorescent controls. Sera containing antibodies for both latent and lytic antigens were considered positives (KSHV^pos^).

We performed molecular genotyping of HLA B (22 alleles), Cw (14 alleles), and KIR (18 genes) by PCR on genomic DNA using sequence specific primers (SSP) according to the manufacturer's instructions (BAG- Lich, Germany, Astra Formedic, Milan Italy). Alleles detection was done after amplification in a GeneAmp PCR 9700 thermocycler (Applied Biosystem, Foster City, CA, USA) by gel electrophoresis on 2% agarose gel.

KIR haplotype and ligands. Two broad haplotypes, termed A and B, were defined based on KIR genes. A and/or B haplotypes were classified based on the criteria adopted by Middleton http://www.allelefrequencies.net[8].

Ligands group was defined as follows:

i. KIRs 2DL1 and 2DS1 bind the C2 epitope (Asparagine at position 77, Lysine at position 80).

ii. KIRs 2DL2, 2DL3 and 2DS2 bind the C1 epitope (Serine at position 77, Asparagine at position 80).

iii. HLA-Bw4 and the HLA-Bw4*80I (Isoleucine at position 80) subset were considered the ligand for KIR 3DL1 and 3DS1 [9,10].

We assessed differences between groups by the chi-square test for categorical variables. P value with Yates correction (p_y_) or Fisher exact test (p_f_) and Bonferroni correction for multiple test (p_c_) were applied. The association of each polymorphism with disease (cKS) was measured by the Odd Ratio (OR) and a 95% Confidence Interval (95%CI). For each independent variable, crude and adjusted odds ratios and 95% confidence intervals were calculated.

Multivariate analysis were performed with binary logistic regression model to assess KIR-HLA genotypes associated with cKS, considering cKS^pos ^versus cKS^neg ^as dependent variable, gender and age as covariates. Analyses were carried out using a SPSS 16.0 for Windows.

KIR gene distribution was evaluated in the three groups studied (Additional file 1: Table S1); no significant difference was detected between KSHV^pos^/cKS^neg ^infected and KSHV^neg ^individuals.

Because distribution of KIR and HLA genotypes were similar between the two control groups, we thought it was appropriate to combine them in subsequent analyses.

**Figure 1:**
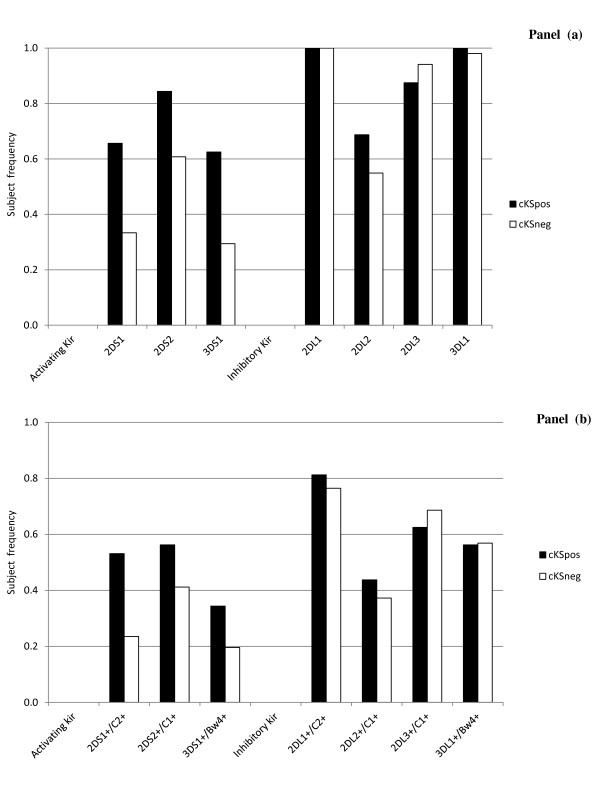
**Panel a: KIR gene distribution in KSHV-infected patients with classic Kaposi Sarcoma (cKS_pos _N = 32), and in age-matched healthy KSHV-infected or uninfected individuals (cKS_neg _N = 51)**. Panel b: Functional KIR-HLA receptor/ligand genotypes in the same individuals. *p = 0.01.

Evaluations focusing on these two groups showed the presence of higher frequencies of activating KIR genes (Figure 1, Panel a) and of activating KIR/HLA receptor/ligand genotypes (Figure 1, Panel b) in KSHV^pos^/cKS^pos ^patients compared to subjects without cKS.

A higher frequency of KIR2DS1+ and KIR2DS1+/C2+ genotypes was detected in cKS^pos ^compared to cKS^neg ^individuals (p_y _= 0.008; OR: 3.82) in KIR2DS1+ which increases to 4,25 in KIR2DS1+/C2+ cKS patients (Table 1). The association between the KIR2DS1+/C2+ genotype and cKS was confirmed after binary logistic regression analysis adjusted for age and gender (*p *= 0.004 OR: 5.2, (95%) IC:1.7-16.3).

**Table 1:** Activating KIRs and Kir/HLA receptor/ligand genotypes distribution in 32 classic Kaposi Sarcoma (cKS^pos^), and in 51 cKS^neg ^(18 KSHV-infected plus 33 KSHV-uninfected) individuals

KIR/KIR-ligands	**cKS**^**pos**^	**cKS**^**neg**^	P value	OR (95%CI)
	N/T %	N/T %		
**2DS1+**	**21/32 (65.6)**	**17/51 (33.3)**	**p_y _= 0.008**	**3.82 (1.4-10.9)**
**2DS1+/C2+**	**17/26 (65.4)**	**12/39 (30.7)**	**p_y _= 0.01**	**4.25 (1.3-14.2)**
2DS1+/C2-	4/6 (66.7)	5/12 (41.7)	p_f _= 0.62	ND
2DS2+	27/32 (84.4)	31/51 (60.8)	p_**y **_= 0.04	3.48 (1.0-12.3)
2DS2+/C1+	18/22 (81.8)	21/38 (56.7)	p_**y **_= 0.09	3.43 (0.9-14.9)
2DS2+/C1-	9/10 (90.0)	10/14 (71.4)	p_f _= 0.36	ND
**3DS1+**	**20/32 (62.5)**	**15/51 (29.4)**	**p_y _= 0.006**	**4.00 (1.4-11.4)**
**3DS1+/Bw4+**	**11/18 (61.1)**	**10/32 (31.2)**	p_y _= 0.08	3.46 (0.9-13.9)
3DS1+/Bw4*80I+	6/11 (54.5)	7/19 (36.8)	p_y _= 0.45	2.06 (0.4-12.3)
**3DS1+/Bw4-**	9/14 (64.3)	5/19 (26.3)	p_y _= 0.68	5.04 (0.9-30.6)
3DS1+/Bw4*80I-	**14/21 (66.6)**	**8/32 (25.0)**	**p_y _= 0.006**	**6.00 (1.5-24.6)**
HLA C2+	26/32 (81.2)	39/51 (76.5)	p_y _= 0.80	1.33 (0.4-4.6)
HLA C1+	22/32 (68.7)	37/51 (72.5)	p_y _= 0.90	0.83 (0.3-2.4)
HLA Bw4	18/32 (56.3)	32/51 (62.7)	p_y _= 0.72	0.76 (0.3-2.1)
HLA Bw4*80I+	11/32 (34.4)	19/51 (37.5)	p_y _= 0.97	0.88 (0.3-2.5)

N/T: Number of subjects/Total subjects; (%) percentage in brackets;: p_y _= p value with Yate's correction,: p_f _= p value calculated with fisher exact test for small number; OR: Odds ratio, IC: Interval of confidence

HLA C2 + (C2/C2; C2/C1), HLA C1 + (C1/C1; C2/C1), HLA Bw4 (Bw4+/Bw4+, Bw4+/Bw4-)

HLA Bw4*80I+ (Bw4*80I+/Bw4*80I+, Bw4*80I+/*Bw4*80I-)

The statistically significant associations are marked in bold

The frequency of KIR3DS1 gene distribution was quite similar to that of KIR2DS1, probably due to their linkage disequilibrium (LD).

Additionally, the activating KIR3DS1+ gene was statistically more frequent in cKS^pos ^compared to cKS^neg ^individuals (p_y _= 0.006; OR:4.00).

HLA-Bw4 molecules are ligands of KIR3DL1 and KIR3DS1, particularly Bw4*80I (bearing an Isoleucine at position 80) [11] showed a stronger binding affinity with KIR3D alleles, therefore both KIR3DS1+/Bw4+ and KIR3DS1+/Bw4*80I + genotypes distributions were analysed (Table 1). However the frequency of KIR3DS1+/Bw4+ as well as KIR3DS1+/Bw4*80I + genotypes in cKS^pos ^compared to cKS^neg ^subjects did not reach the statistical significance (p_y _> 0.05). Conversely, KIR3DS1+/Bw4*80I- frequency resulted still statistically higher in cKS than in controls (p_y _= 0.006; OR:6.00), confirming that the KIR3DS1 positivity, but not the KIR/HLA receptor/ligand combination 3DS1/Bw4*80I, was associated to cKS.

Finally KIR2DS2 gene and KIR2DS2/C1+ genotypes were quite equally distributed in all the studied groups.

The frequencies of HLA ligands: HLAC2 + (C2/C2; C2/C1), HLA C1 + (C1/C1; C2/C1), HLA Bw4+ (Bw4+/Bw4+, Bw4+/Bw4-) and Bw4*80I + (Bw4*80I+/Bw4*80I+, Bw4*80I +/Bw4*80I-) are shown in Table 1. No statistical differences were observed between groups, revealing that differences observed for KIR 2DS1+/C2+ was likely due to the KIR/HLA receptor/ligand interaction and not to either KIR or to HLA genes separately.

Finally, no differences were observed in the genotype distribution of the other examined inhibitory and activating KIR genes (Additional file 1: Table S1) or in the frequency of HLA-B and HLA-Cw alleles (not shown).

In this work we investigated whether specific KIR genes and their HLA class I ligands would be associated with classic Kaposi sarcoma in KSHV^pos ^infected subjects. Results herein, albeit stemming from analyses performed in a limited number of individuals, indicate that a statistically higher frequency of activating KIR genes together with their HLA ligands is present in KSHV-infected patients who have cKS compared to individuals including those with KSHV infection without cKS

Importantly, whereas activating KIR genes were more frequent in cKS patients, the distribution of KIR genes was similar between KSHV^pos ^and KSHV^neg ^healthy controls; this allowed us to combine them in a single control group. The KIR2DS1+ and KIR3DS1+ genes were prevalent in cKS patients; This similarity of distribution may be secondary to the fact that these genes are in linkage disequilibrium. However, whereas the activating KIR2DS1+/C2+ genotype was positively associated with cKS development, HLA ligand distribution did not reveal any statistical association with cKS. This observation, together with the fact that the OR of KIR2DS1 association increased when the KIR2DS1+/C2+ genotype was evaluated, may suggest that the association of KIR2DS1+/C2+ with cKS is mostly due to KIR-HLA interaction and not to either KIR or to HLA genes separately.

Data herein indicate that a KIR/HLA "activating milieu" is present in cKS; such milieu may be a risk factor for the development of this tumor. Our results add cKS to the list of virus-associated cancers including cervical neoplasia [12,13] and nasopharyngeal carcinoma [14] where an increased presence of activating KIR/HLA receptor/ligand combinations is observed and is suggested to play a role in oncogenesis.

Genotypes that increase the activation of NK cells are possibly beneficial in viral infections such as HIV and HCV [15]; inflammatory components associated with NK cell activation might nevertheless have a detrimental effect, possibly facilitating the oncogenic process for cancers that, as is the case with cKS, have an inflammatory component [11,16]. Thus, the elicitation of inflammation and NK cells activity via KIR/HLA interactions might be directly involved in malignant transformation and tumor progression as a consequence of the production of cytokines such as TNF-α, IL-6, and TGF-β that contribute to the proliferation and survival of tumor cells [17]. Non-specific inflammatory responses, such as oxidative DNA damage, may be triggered by activated NK upon KIR-HLA interaction as well, possibly facilitating the growth of oncogenic viruses [18].

The small sample size is a significant limitation of this study. We tried to bypass this problem by sampling together KSHV-infected and -uninfected healthy individuals, once we verified that the distribution of KIR and HLA molecules was comparable.

It is important to underline that these data stem from analyses performed in a relatively small group of individuals and will need to be validated using bigger cohorts of KS patients.

## Competing interests

The authors declare that they have no competing interests.

## Authors' contributions

FRG and RM analyzed and interpreted data, FRG drafted the manuscript. SA, CA, MZ, AH and MGC performed serological assays and molecular HLA and KIR genotyping. AT, MB and LB enrolled patients and gave clinical notices. FRG, MB and SA interpreted data and edited the paper, MC conceived the idea, guided data analysis, interpreted data and edited the paper. All authors had access to data, commented on and contributed to the final draft of the manuscript. All authors read and approved the final paper

## Supplementary Material

Additional file 1 **Table S1**. Inhibitory and activating KIRs genotype distribution in 32 classic Kaposi sarcoma (cKS^pos^), 18 KSHV-infection without Ks (KSHV^pos^/cKS^neg^) and in 33 KSHV-uninfected individuals (KSHV^neg^). Click here for file
