# Supplementary material for: Activating KIR/HLA complexes in classic Kaposi's Sarcoma
Source: Infect Agent Cancer. 2012 Apr 2;7:9. doi: 10.1186/1750-9378-7-9 (PMC3379936; doi:10.1186/1750-9378-7-9)
Supplement: Additional file 1 — Table S1. Inhibitory and activating KIRs genotype distribution in 32 classic Kaposi sarcoma (cKSpos), 18 KSHV-infection without Ks (KSHVpos/cKSneg) and in 33 KSHV-uninfected individuals (KSHVneg). [file 1750-9378-7-9-S1.DOC]

**Supplementary Table 1: inhibitory and activating KIRs genotype** distribution in 32 classic Kaposi sarcoma (cKS^pos^ ), 18 KSHV-infection without Ks (KSHV^pos^ /cKS^neg^) and in 33 KSHV-uninfected individuals (KSHV^neg^).

| **KIR** | **cKS^pos^** | | **KSHV^pos^ /cKS^neg^** | | **KSHV^neg^** | |
| --- | --- | --- | --- | --- | --- | --- |
|  | N | % | N | (%) | N | (%) |
| Activating KIR |  |  |  |  |  |  |
| 2DL4 | 32 | (1,00) | 18 | (1,00) | 32 | (97,0) |
| 2DS1 | **21^1^** | **(65,6)** | 6 | (33,3) | **11^1^** | **(33,3)** |
| 2DS2 | **27^2^** | **(84,4)** | 13 | (72,2) | **18^2^** | **(54,5)** |
| 2DS3 | 18 | (56,3) | 5 | (27,8) | 10 | (30,3) |
| 2DS4*001/2 | 7 | (21,9) | 3 | (16,6) | 2 | (6,10) |
| 2DS4*003/7 | 25 | (78,1) | 14 | (78,7) | 30 | (90,9) |
| 2DS5 | 14 | (43,8) | 4 | (22,2) | 7 | (21,2) |
| 3DS1 | **20^3,4^** | **(62,5)** | **5^3^** | **(27,8)** | **10^4^** | **(30,3)** |
| Inhibitory KIR |  |  |  |  |  |  |
| 2DL1 | 32 | (1,00) | 18 | (1,00) | 33 | (1,00) |
| 2DL2 | 22 | (68,8) | 10 | (55,5) | 18 | (54,5) |
| 2DL3 | 28 | (87,5) | 18 | (1,00) | 30 | (90,9) |
| 2DL5a | 12 | (37,5) | 2 | (11,1) | 10 | (30,3) |
| 2DL5b | 7 | (21,9) | 2 | (11,1) | 5 | (15,2) |
| 3DL1 | 32 | (1,00) | 17 | (94,4) | 33 | (1,00) |
| 3DL2 | 32 | (1,00) | 18 | (1,00) | 33 | (1,00) |
| 3DL3 | 32 | (1,00) | 18 | (1,00) | 32 | (97,0) |

(KSHV^pos^ /cKS^pos^) patients, 18 KSHV positive healthy controls (KSHV^pos^/cKS^neg^) and in 33 healthy Controls (KSHV^neg^ ). N: Number of subjects; (%) percentage in brackets; p_y_=p value with Yate’s correction, : p_f_, =p value calculated with fisher exact test for small number; OR: Odds ratio, IC : Interval of confidence.

^1^ **KSHV^pos^/cKS^pos^** vs **KSHV^neg^** p**_y_**=0,02; OR: 3,82 , 95%CI:1,2-12,3

^2^ **KSHV^pos^/cKS^pos^** vs **KSHV^neg^** p**_y_**=0,02; OR: 4,50, 95%CI:1,2-17,4

^3^ **KSHV^pos^/cKS^pos^** vs **KSHV^pos^/cKS^neg^** p**_y_**=0,04; OR: 4,33, 95%CI:1,1-18,6

^4^**KSHV^pos^/cKS^pos^** vs **KSHV^neg^** p**_y_**=0,02; OR: 3,83 , 95%CI:1,2-12,4
